# Supplementary material for: Strategies for Improving the CO2 Adsorption Process of CPO-27-Mg through Thermal Treatment and Urea Functionalization
Source: Materials (Basel). 2022 Dec 22;16(1):117. doi: 10.3390/ma16010117 (PMC9821002; doi:10.3390/ma16010117)
Supplement: Supplementary file 1 [file materials-16-00117-s001.zip › materials-2063405-supplementary.pdf]

Mass Spectrum

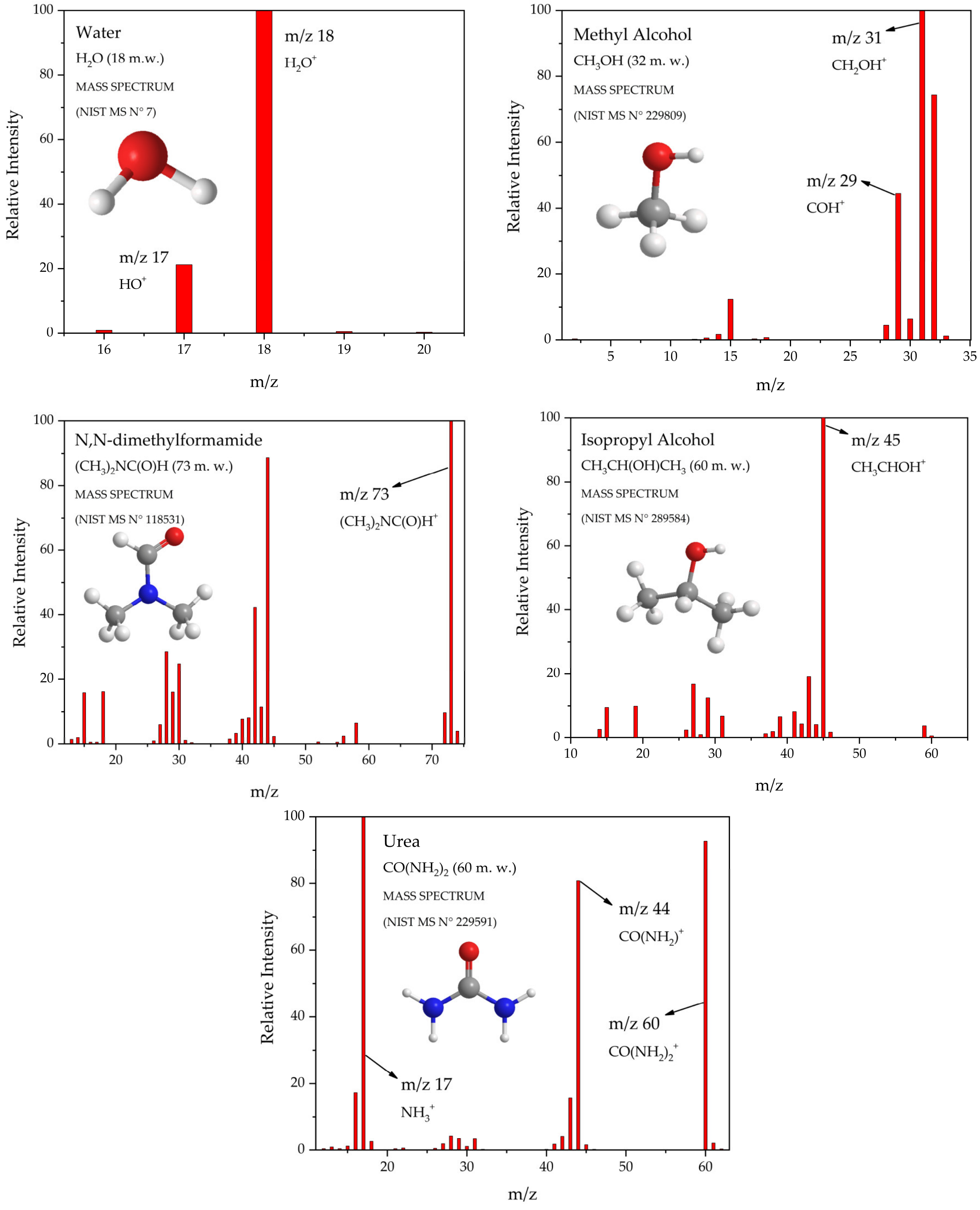

Figure S1. Mass Spectrums.

TG-MS F10

F10

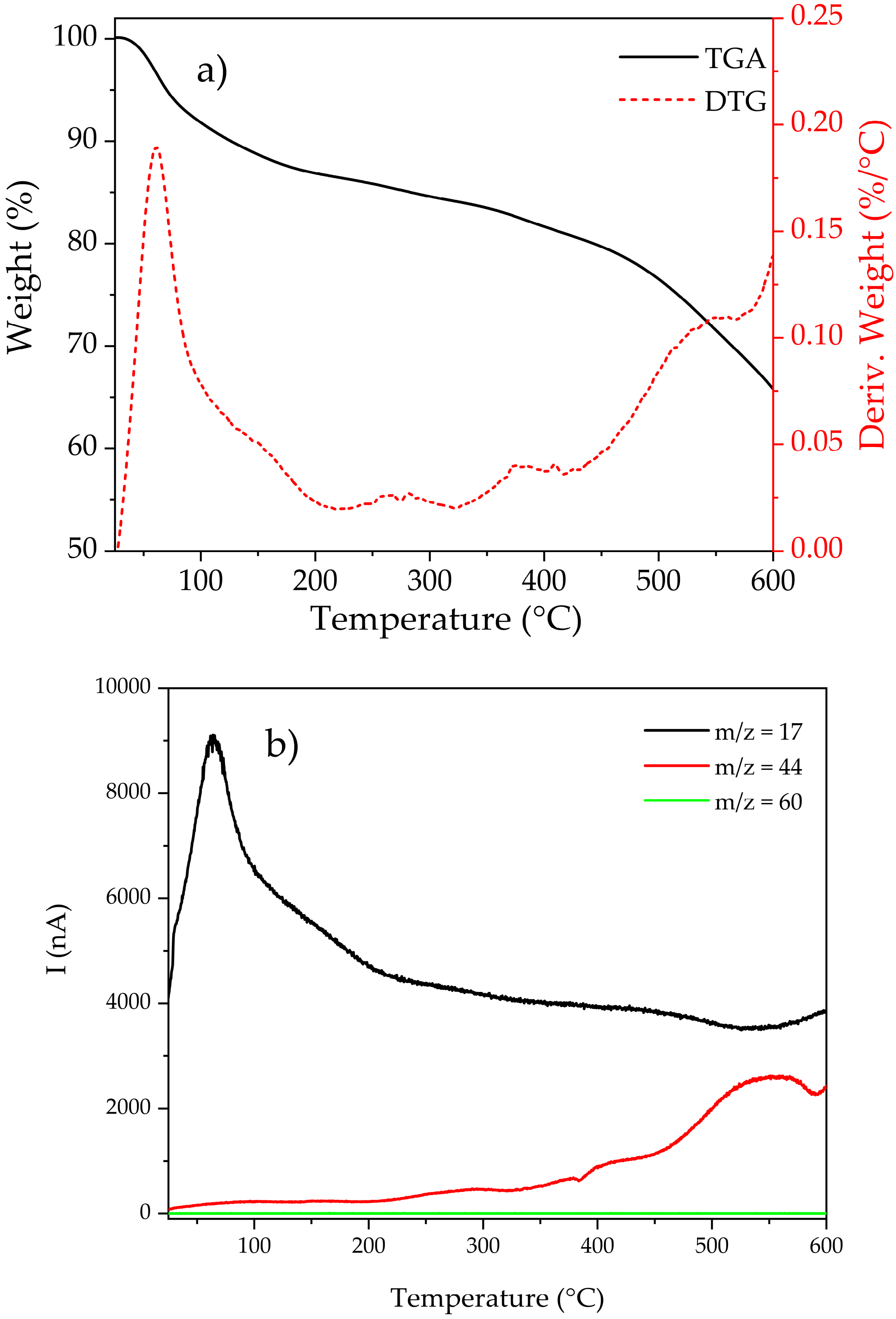

Figure S2. (a) TG and (b) MS analysis (m/z 17, 44 and 60) of **F10**.

TG-MS F25

F25

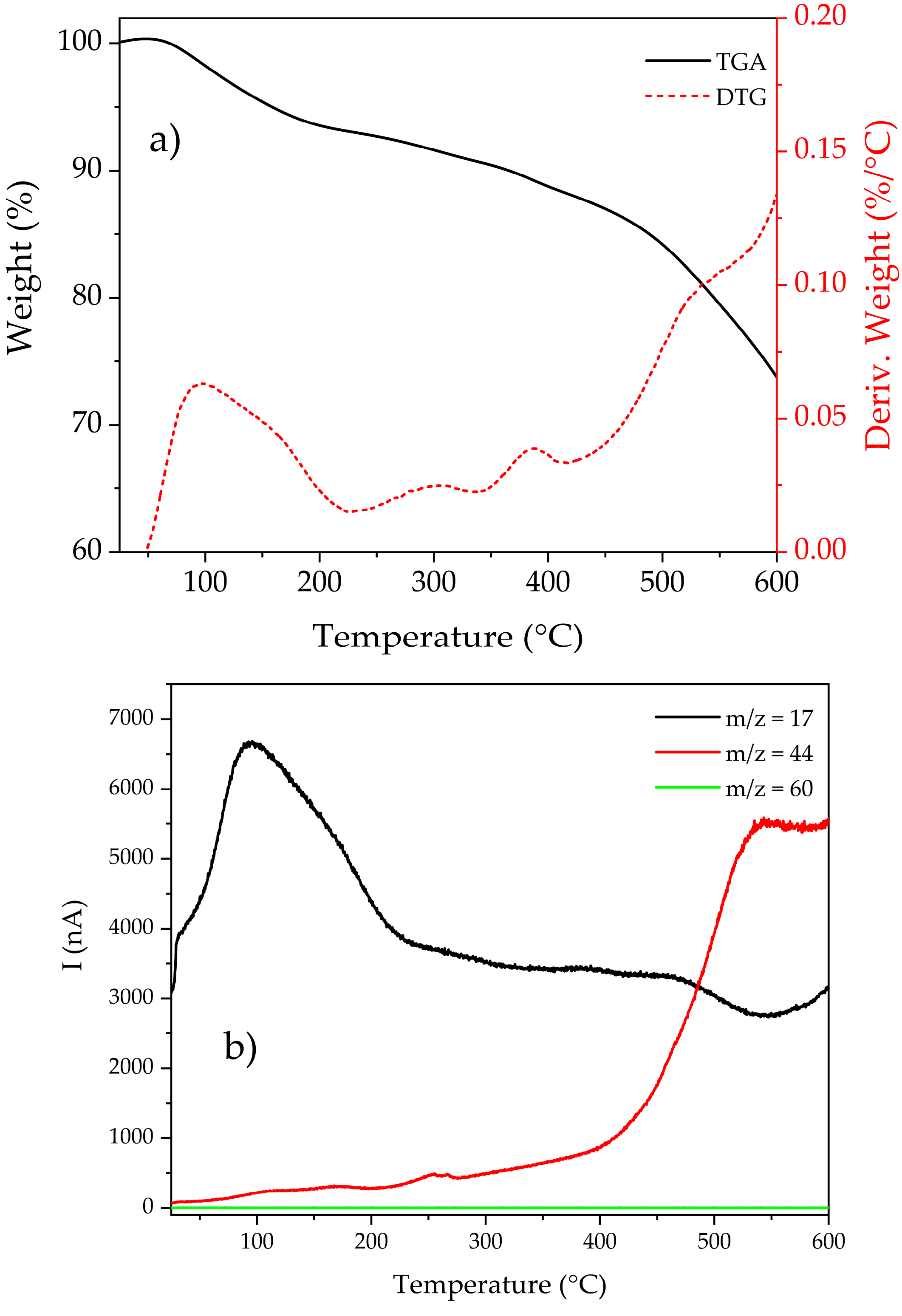

Figure S3. (a) TG and (b) MS analysis (m/z 17, 44 and 60) of **F25**.

TG-MS F50

F50

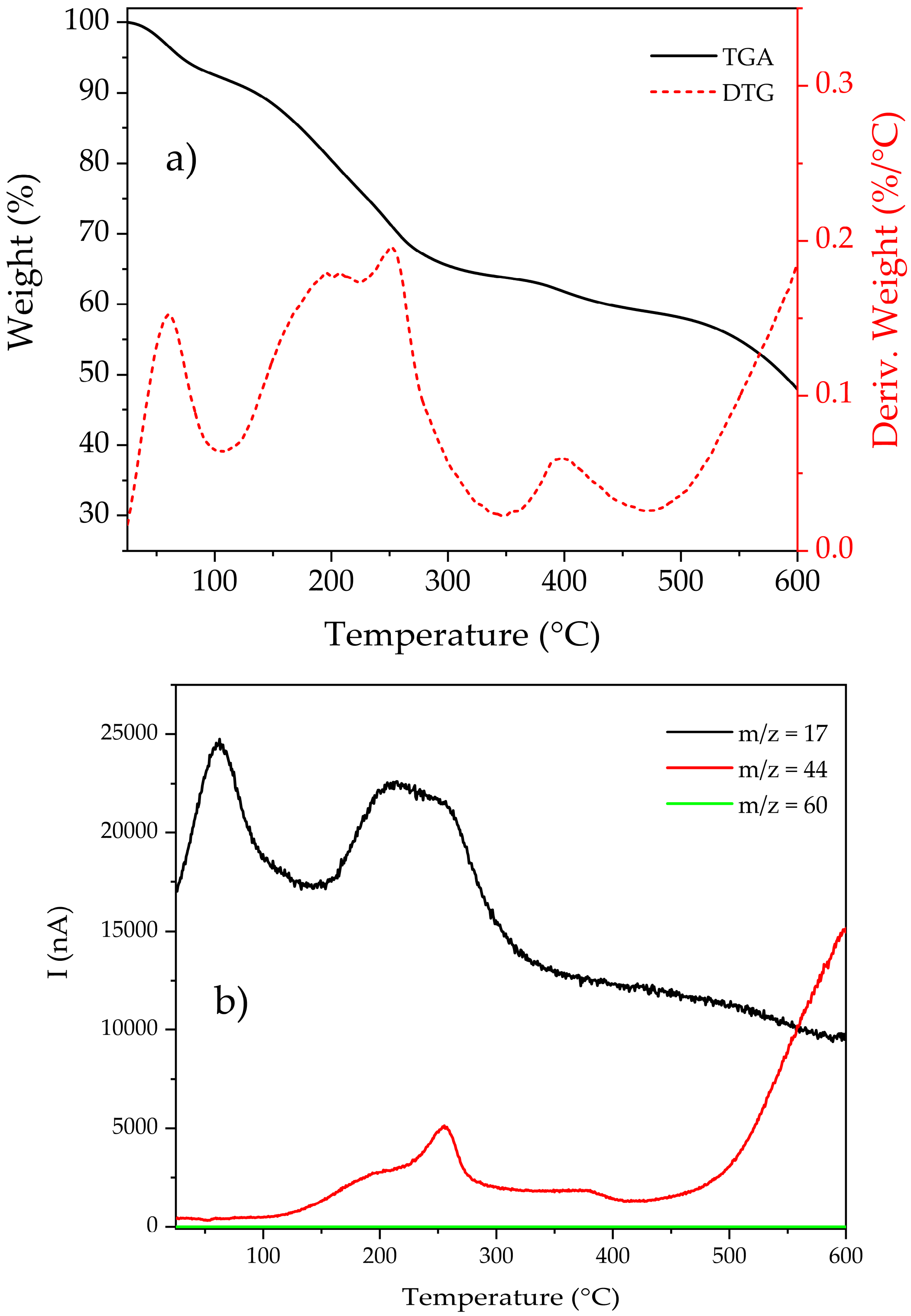

Figure S4. (a) TG and (b) MS analysis (m/z 17, 44 and 60) of **F50**.

TG-MS F100

F100

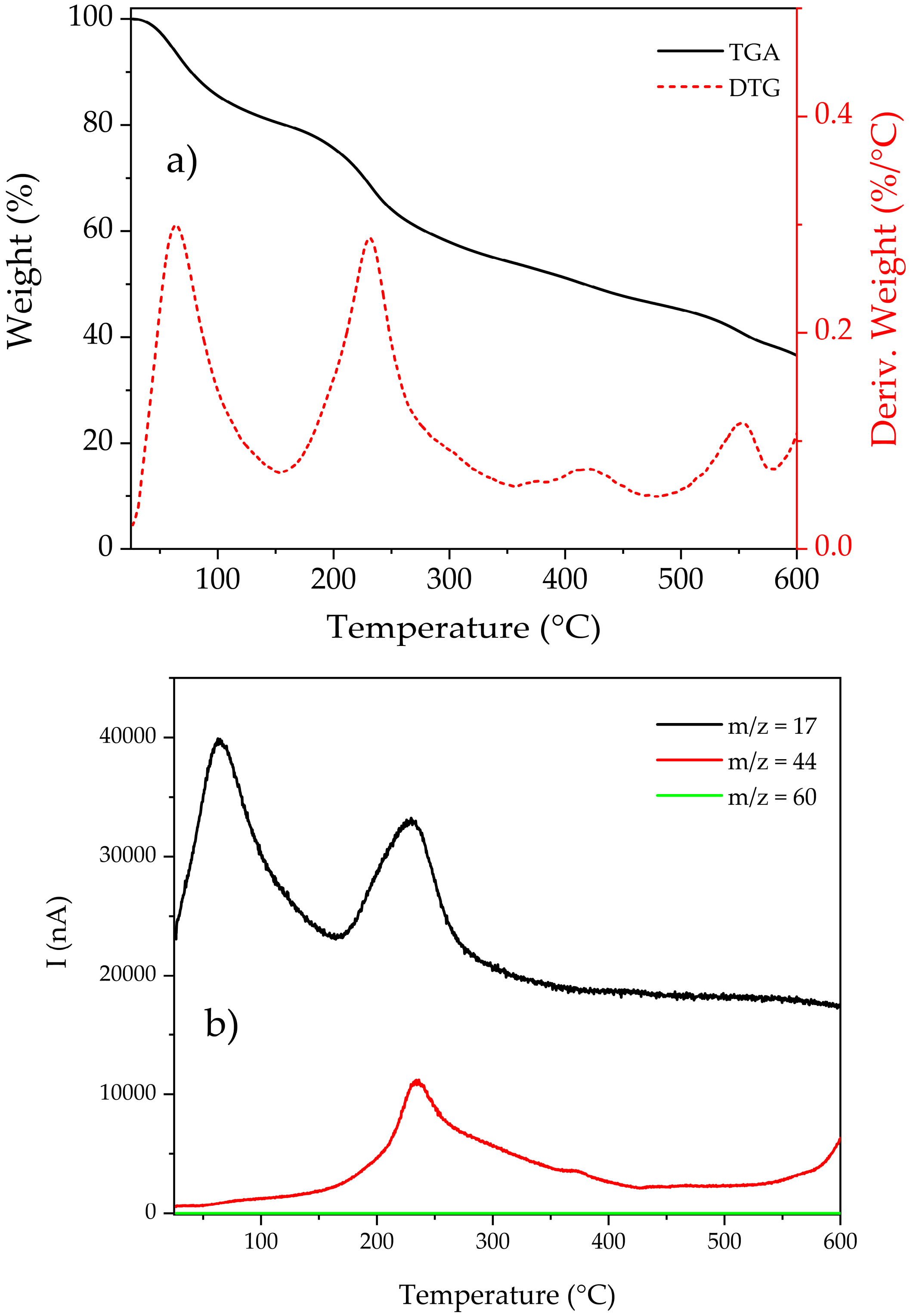

Figure S5. (a) TG and (b) MS analysis (m/z 17, 44 and 60) of **F100**.
